# Invertebrate Biosecurity Challenges in High-Productivity Grassland: The New Zealand Example

**DOI:** 10.3389/fpls.2016.01670

**Published:** 2016-11-15

**Authors:** Stephen L. Goldson, Barbara I. P. Barratt, Karen F. Armstrong

**Affiliations:** ^1^Bio-Protection Research Centre, Lincoln UniversityCanterbury, New Zealand; ^2^Biocontrol and Biosecurity Group, AgResearchCanterbury, New Zealand; ^3^Biocontrol and Biosecurity Group, AgResearchOtago, New Zealand

**Keywords:** pastoral, invasive species, hitchhiker, quarantine, border biosecurity, biosecurity risk

## Abstract

To protect productive grasslands from pests and diseases, effective pre- and at-border planning and interventions are necessary. Biosecurity failure inevitably requires expensive and difficult eradication, or long-term and often quite ineffective management strategies. This is compared to the early intervention more likely for sectors where there is public and political interest in plants of immediate economic and/or social value, and where associated pests are typically located above-ground on host plantings of relatively limited distribution. Here, biosecurity surveillance and responses can be readily designed. In contrast, pastures comprising plants of low inherent unit value create little, if any, esthetic interest. Yet, given the vast extent of pasture in New Zealand and the value of the associated industries, these plants are of immense economic importance. Compounding this is the invasibility of New Zealand’s pastoral ecosystems through a lack of biotic resistance to incursion and invasion. Further, given the sheer area of pasture, intervention options are limited because of costs per unit area and the potential for pollution if pesticides are used. Biosecurity risk for pastoral products differs from, say, that of fruit where at least part of an invasive pathway can be recognized and risks assessed. The ability to do this via pastoral sector pathways is much reduced, since risk organisms more frequently arrive via hitchhiker pathways which are diffuse and varied. Added to this pasture pests within grassland ecosystems are typically cryptic, often with subterranean larval stages. Such characteristics make detection and response particularly difficult. The consequences of this threaten to add to the already-increasing stressors of production intensification and climate change. This review explores the unique challenges faced by pasture biosecurity and what may be done to confront existing difficulties. While there is no silver bullet, and limited opportunity pre- and at-border for improving pasture biosecurity, advancement may include increased and informed vigilance by farmers, pheromone traps and resistant plants to slow invasion. Increasingly, there is also the potential for more use of improved population dispersal models and surveillance strategies including unmanned aerial vehicles, as well as emerging techniques to determine invasive pest genomes and their geographical origins.

## Introduction

Biosecurity, as described broadly by the FAO (2003), is a holistic process that seeks to manage biological risks associated with food and agriculture, not the least of which are invasive alien invertebrates. As in all agricultural sectors, the threat of arrival and establishment of pasture pests will only soar in the future as drivers such as trade, travel, and climate change continue to intensify and diversify. Climate change is already linked to the extending distribution of some pest species in response to warmer temperatures (FAO, 2008).

The international importance of biosecurity is widely recognized (e.g., Barlow and Goldson, 2002; Waage and Mumford, 2008) and prevention of pest establishment is key to effective biosecurity (Simberloff et al., 2013). To this end, the benefits of stringent risk assessment (Keller et al., 2007) and operational prevention of arrival, early detection, and eradication are feasible for many land-based industries. However, New Zealand’s high-performance, improved pastures present their own unique and demanding biosecurity challenges. There the country’s cost-effective pastoral farming methods are based on year-round production of high-producing grass and clover varieties. While such simple ecosystems create great value, they are also almost uniquely vulnerable to invasive pests and diseases (e.g., Goldson et al., 2014a) and, based on the current pest burden, around NZD $1 billion dollars’ worth of production loss and costs are already being incurred by the sector (e.g., Goldson, 2014). This contribution reviews reasons why generally effective biosecurity strategies in other sectors are particularly challenged in pastures and comments on what may be done to deal with the threat of invasive pest species in the future.

## The Existing New Zealand Biosecurity System

**FIGURE 1:**
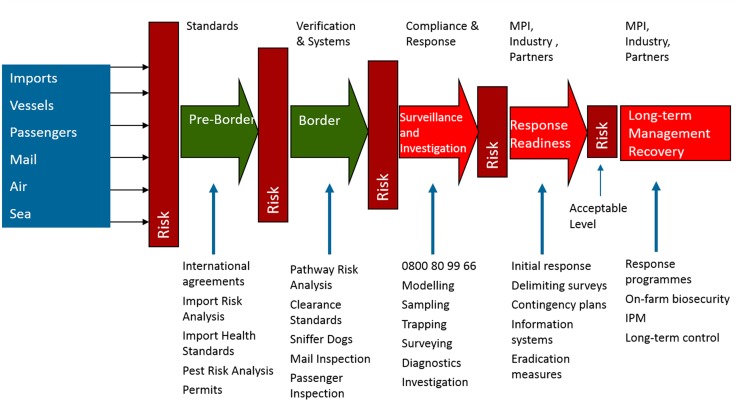
**The structure of New Zealand biosecurity depicted by MPI as a series of pre-, at-, and post-border systems and activities that are designed to successively reduce the risk from incoming sources of exotic pests, and ultimately minimize the need for long-term management**.

New Zealand has developed one of the most comprehensive agricultural biosecurity systems in the world (**Figure 1**). This has arisen as a consequence of the significant dependence of the country on peerless primary production exports and vulnerability to invasive species. Also the country has the advantage of comprising distant islands, and hence borders that are more defensible than those found in jurisdictions within large regions.

For New Zealand, the present biosecurity model shows activity pre-, at-, and post-border (**Figure 1**). Initial pre-border stages deal with threats at their place of origin. Thus for the importation of goods, evidence is required from the exporter demonstrating that offshore biosecurity requirements have been fulfilled, including compliance with any specifically designated product import health standards. These pre-border actions are often supplemented by pathway risk analyses in conjunction with physical interventions at the border itself, such as passenger baggage, vehicle and container inspection. This may include use of detection systems such as sniffer dogs and X-ray. The information gained based on actual interceptions is fed into ever-developing sophisticated risk models designed to assist in decision-making and determination of the biosecurity requirements associated with various types of freight (e.g., Jamieson et al., 2013). A large part of such effort assumes that the target threats and their places of origin are as reasonably well known as possible, for example, with fruit or wood product imports. The benefit here is that it provides the opportunity to monitor and manage recognized pest threat pathways known to be associated with the imported biological products. In turn this facilitates threat interception and disinfestation through treatments such as pesticides, heat-treatment or washing.

Issues around passenger arrivals have been well-managed via baggage X-rays, sniffer dogs, and passenger profiling (Ministry for Primary Industries [MPI], 2013a), but there remains the vast challenge posed by the arrival each year of c. 600,000 containers, 90,000 used vehicles and machinery and 17,000,000 tonnes of cargo (Ministry for Primary Industries [MPI], 2013b). This huge task is variously tackled by the presentation of bills of lading, pathway risk-profiling, use of transitional unloading facilities, and employment of trained accredited inspectors. Irrespective, hitchhiker pests continue to arrive via this route, including insects that are attracted to port and ship lights and/or arrive as contaminants of plant or soil material on inanimate objects (McNeill et al., 2011; Hulme, 2015). A close container inspection survey has revealed that of c.1000 containers landed at the main centers in New Zealand c.13% carried potentially threatening contaminants (Gadgil et al., 2002).

## The Unique Challenge of Pastoral Biosecurity

The value of the pastoral sector to New Zealand is very significant at c. NZD$24.3 billion p.a. and as such contributes >40% of the country’s merchandisable exports^1^. The base for this comprises high-producing ryegrass and clover varieties that are well attuned to the country’s favorable climate and these plants contribute NZD17.2 billion p.a. to the national GDP (NZIER, 2015). Improved pasture now occupies c. 10.6 m ha in New Zealand (c. 40% of the total land area)^2^ and about a third of this consists of intensively managed sward of mainly ryegrass/clover. From virtually pest-free origins in the 19th century, these pastures have now acquired a significant burden of exotic pest species; Barlow and Goldson (2002) noted that 90% of the country’s pasture pest species are exotic. The most damaging of these include the clover root weevil (*Sitona obsoletus* Gmelin), the Argentine stem weevil (*Listronotus bonariensis* Kuschel), the lucerne weevil (*S. discoideus* Gyllenhal) and the blue green aphid (*Acyrthosiphon kondoi* Shinji; Goldson et al., 2005). Further, African black beetle (*Heteronychus arator* Fabricius) is causing increasing damage in the North Island partly as a result of climate change. In Australia, it has been observed that with the rapid expansion in improved pastures since the 1950s there was a widespread decline in productivity of pasture legumes in the1970s and 1980s. This is considered, in part, to have been due to the occurrence of new insect pests (Wolfe, 2009). Undoubtedly, the clover root weevil has had a similar effect in New Zealand (e.g., Gerard et al., 2007).

The reasons for the severity of impact of such invasions are undoubtedly varied. For many years the New Zealand pastoral sector has been confronted with the need to pursue efficiency largely through intensification. This has resulted in elite pasture grasses and clovers being bred for traits that offer enhanced agronomic performance, but which have tended to make them more susceptible to pest damage. Significantly, however, Goldson et al. (2014a,b) have contended that New Zealand’s ryegrass/clover-dominant pastoral ecosystems are notable for their lack of invertebrate biodiversity. Irrespective of whether this is strictly correct (and work is now in progress to examine this), New Zealand pastures, that comprise partial transplants of complex systems found elsewhere, are unlikely to include the same diversity of key exotic pest-suppressing species such as parasitoids, generalized predators and predatory spiders, as occur in the invasive pests’ native ranges. This is in spite of many of New Zealand’s pastures superficially resembling the large grassland areas found elsewhere (e.g., forb-rich European meadows). More generally, it is thought that it is this lack or difference of complexity that renders New Zealand’s improved grasslands extremely susceptible to invasive exotic species (Goldson et al., 2005) and, as such, is typical of island ecosystems generally (e.g., Reaser et al., 2007). When invaders enter New Zealand pastures they encounter abundant food supply, unfilled niches, and a lack of the biotic resistance often found elsewhere in the form of interacting guilds of natural enemies and diseases (Tylianakis and Romo, 2010). This similarly applies to the functional diversity in the hedgerows and headlands of New Zealand’s farmed ecosystems; again there is less exotic pest suppression capability than found in the equivalent ecosystems in the native range. It is this ecological setting that has led to the spectacularly high and damaging populations of invasive pest species that stabilize at far greater densities than those found in the native ranges (Goldson et al., 2014b). The impact of this scenario is certainly exacerbated by how very easily overlooked the damage is, for example by Argentine stem weevil, with its negative effects being attributed to poor seed germination and drought, as well as the impact of other plant stresses. Overlooking these negative factors more than anything else, has also made attainment of sustainable funding difficult for research projects to address the problem.

The incontrovertible importance of pastoral production to New Zealand and the continuing accumulation of destructive pests have resulted in an urgent need for excellent, effective, and robust pasture biosecurity measures. Unfortunately, actual biosecurity threats to pastures tend to get relatively sparse mention compared to the impacts of existing pests, e.g., in Australia (Wolfe, 2009) and in the UK (Clements, 1980; Hopkins, 2008). Often references to modified grasslands are focused more on amenity turf rather than grazed rangeland and meadow systems. For example, in the United States Vittum et al. (1999) published a comprehensive compendium of turfgrass insects of the United States and Canada. Potter and Held (2002) subsequently noted that the Japanese beetle, *Popillia japonica* Newman, an introduced scarab, had become the most widespread and destructive insect pest of turf, landscapes and nursery crops in the eastern United States. Indeed, until recently, even in New Zealand there has been a preoccupation with managing the current pest loading rather than tackling pre-establishment biosecurity *per se* (Moot et al., 2009).

The reasons for this comparative low level of focus on pasture biosecurity are undoubtedly varied. In some ways, and despite reality, pasture is not viewed by the public as a particularly valuable ‘crop’. Compared to say kiwifruit or apples, pasture plants are seen to be of low inherent unit value with not much esthetic appeal (Goldson et al., 2005). Related to this, cosmetic pest damage to forage is absolutely unimportant since forage itself is not exported. Further, many pasture pests are well-camouflaged against pasture soil and surrounding litter. Paradoxically, the light colored root-feeding subterranean early stages in the soil are sometimes easier to detect visually than the adults, although this does require turning the soil which is a further hindrance. However, even the advantage of exposure is offset by their typically indistinct morphology which limits taxonomic resolution to only a few species (AgPest^3^). Another consideration is that although these exotic pest species frequently cause severe pasture plant damage, it may only become visually apparent at certain times of the year, typically during peak spring and autumnal growth. This frequently leads to misidentification of the problem (e.g., poor seed quality, drought, etc.).

The subterranean habit of larval stages of many pasture pest species makes eradication of new invasive species nearly impossible once populations have established beyond a few hectares, e.g., clover root weevil, black beetle, and tropical grass webworm (Barker et al., 1996**)**. Added to this, is the extensive distribution of pasture that determines low rates of economic return per hectare and precludes many surveillance and intervention options.

Significantly, pasture biosecurity also presents less opportunity for pathway-based biosecurity intervention (**Figure 1**) such as can be implemented in high value imports and export chains. Rather, a recent assessment of exotic pests that could be hazardous to New Zealand pasture in the future pointed to the primary importance of difficult-to-manage hitchhiker pathways including containers, used agricultural machinery and passengers (Toy, 2013). In general, adventive hitch-hiker species are most likely to be recognized and dealt with as part of the ongoing risk profiling and disinfestation processes in other pathways and for other agricultural sectors.

All of these factors make the New Zealand biosecurity situation for pastures both different and difficult.

## Prospects for Improved Pasture Biosecurity

Given the critical importance and the vulnerability of the New Zealand pastoral sector to biosecurity failure, it is necessary to consider how a biosecurity system may be developed further to suitably accommodate the unique needs and challenges outlined above. Certainly, part of the existing suite of techniques and technologies already being applied in general to New Zealand border biosecurity will benefit pasture biosecurity. However, the question is whether and how this can be more specifically extended and augmented. There are a number of areas that merit consideration.

### Pest Proofing of Pastures

Waage and Mumford (2008) have suggested that there is value in creating resilience to invasion into agroecosystems rather than ‘building walls’. This is particularly applicable to pasture because of its sheer invasibility. Thus, part of any evolving biosecurity strategy for pasture could include improving resilience to pest establishment and dispersal through pasture diversification (Sanderson et al., 2013) or plant resistance. In New Zealand an enormous advance occurred in pest-proofing pastures with the discovery and adoption of naturally occurring obligate biotrophic endophytic fungi (*Epichloë* spp.). This severely suppresses or controls pests of ryegrass and tall fescue (Johnson et al., 2013) and would be anticipated to be very useful in imparting resistance to any new exotic pests. Consequently, as pasture in New Zealand still essentially comprises ryegrass and white clover, continued advancement of this endophyte technology would provide an extremely useful barrier to establishment of new pests should they arrive, and thus contribute conspicuously to biosecurity for the sector as a whole. There has also been success in breeding lucerne (*Medicago sativa* L.) for resistance to aphids (Barlow and Goldson, 2002). Similarly, there is the ability to introduce new plant material (Wolfe, 2009) that may enhance existing generic biocontrols (Vattala et al., 2006). Likewise approaches could be taken to pest-proof pasture soil by manipulating the microbial ecology such that it is less acceptable to the soil-dwelling stages of some invasive species (Ferguson et al., 2012). Finally, while currently not permissible in New Zealand for societal and export reasons, the creation of new forms of resistance is possible through host plant genetic manipulation.

### Industry and Farmer Awareness

Within the pastoral sector the ongoing invasion throughout New Zealand by the clover root weevil, *S. obsoletus*, has certainly raised awareness of the need for biosecurity (Basse et al., 2015). Linked to this there is real opportunity for pastoral biosecurity to advance on the basis of a strong social component (citizen science) whereby farmers in particular maintain a high level of biosecurity vigilance. This requires ready access to relevant information and data sources such as AgPest^1^, as well as to the appropriate government agency (in New Zealand, MPI). Important to this also are the industry organizations groups such as Dairy New Zealand^4^, Beef + Lamb New Zealand^5^ and the Foundation for Arable Research^6^. These organizations play an essential role in raising suitable awareness and provide ready access to industry networks. In this respect the New Zealand Government Industry Agreement^7^ (GIA) process is likely to be valuable. Such opportunities in the near-term are highly likely to involve increasingly sophisticated and widespread rapid access to information via hand-held electronic devices, most likely smart phones.

### The Use of Lists and Data Bases

There are a number of important reference sites for New Zealand biosecurity. These include, the International Plant Sentinel Network, IPSN^8^ which has a focus on linking botanic gardens, National Plant Protection Organizations (NPPOs) and the work of various plant health scientists’ associations. All of these can provide early warning systems of new and emerging pest and pathogen risks, including pasture and turf plant species. Further, initiatives such as The Biological Heritage National Science Challenge^9^ in New Zealand, which seek to develop in depth knowledge of what species are already in the country, will provide a more solid foundation from which to recognize new species incursions. Also the Global Eradication and Response Database (GERDA) (Kean et al., 2016) aims to summarize all incursion response and eradication programs from around the world to share experience and enhance opportunities for future biosecurity responses.

In general terms, an immediate component of heightened pasture biosecurity is the identification of species with potential high impact and likelihood of arrival and establishment in New Zealand. Traditionally, this response has been to compile lists from evidence offshore of those insect species known to be damaging. Such lists are both logical and useful although they can be of mixed value if adhered to too rigidly to the species level. Irrespective, ranking of which exotic species could be a threat to New Zealand pasture can give very important broad indications of what to look out for. For example, based on the combined potential to establish and have an adverse impact, a recent report named 151 potential hazards (Toy, 2013). With reference to the ability to establish, 24 species were highly rated. Of these, 22 were insects, nine of which were Coleoptera; of the others, Diptera, Hemiptera, and Lepidoptera were represented. However, only seven of the 22 were rated high in both their establishment potential and probable impact potential. Four of these were Coleoptera, viz. *Agriotes sputator* (Linnaeus), *Hypera postica* (Gyllenhall), *S. hispidulus* (Fabricius), and *Sphenophorus venatus* (Say). Thus root-feeding beetles could be considered the most obvious group to look out for. Certainly this aligns well with New Zealand experience to date. Five of the nine severe pasture pest arrivals that have occurred since trade intensity increased in the 1920s have been Coleoptera and all are likely to have arrived as hitchhikers. It is salutary to note that there are c.100 *Sitona* spp. in the Palaearctic region (Velazquez de Castro et al., 2007), most of which have the ability to damage forage legumes and all of which are very difficult to recognize in the field as separate species (Phillips and Barratt, 2004). Interpreting lists as above also permits the cataloging of those traits and life histories that are indicative of ‘types’ of species that need to be prioritized as potential biosecurity threats, such as root feeding Scarabidae, rhizobial nodule feeding Curculonidae, phloem-feeding Aphidae, and vascular feeding Pentotomidae.

Obviously, the ability to classify hitchhiker pathways known to be associated with greater risk need to be advanced in terms of the taxa, goods and seasonality correlates. For example, southern hemisphere countries are sources of Coleoptera in the same lifecycle phase which makes establishment more likely. Evidence shows that Australia is a particular risk for New Zealand partly because of its geographic proximity, the survivability of the insects during brief transport and synchrony of seasons.

### Emerging Technologies

Technology, albeit slowly, is increasing its capacity to assist pastoral biosecurity. It follows that improved surveillance would most usefully be focused in the vicinity of seaports, airports, rail routes, large transitional facilities, and tourist centers. There are a number of existing and new opportunities to enhance the chances of detection of exotic threats to pastures. None of these singularly suggest a breakthrough, but collectively these technologies may be brought to bear along with enhanced passive surveillance by the New Zealand community, particularly pastoral farmers.

More specifically, there is the possibility of enhanced use of ‘sentinel or trap plants’ that can be examined regularly to more clearly indicate the presence of a new species. Such an approach has been successfully used as a method for identifying potential pests found in off-shore pasture ecosystems or in plant collections such as botanic gardens (Roques et al., 2015). China is New Zealand’s largest trading partner where there are extensive areas of pasture in similar climatic zones to those in New Zealand. Examination of pests and diseases attacking pasture plants in that country could point to potential pests that could arrive with large volumes of trade. Direct trapping can also offer enhanced detection; ‘delta’ sticky traps baited with *S. lineatus* synthetic aggregation pheromone have been shown to catch high numbers of various *Sitona* spp. in the vicinity of lucerne crops (Toshova et al., 2009). Similarly, smart traps for lepidopteran and dipteran pests have been shown to be effective (Liu et al., 2009). For example pheromonal lures have been useful in dealing with an outbreak of an Australian pasture tunnel moth (*Philobota* sp.) in northern New Zealand pasture (Dymock et al., 2009).

There are some novel approaches emerging that will be useful for all sectors. Very preliminary work has investigated the potential to detect hitch-hiker pests in confined spaces such as shipping containers, based on the detection of organic volatile compounds known to be associated with pest threats (More et al., 2007). Rapid field-based diagnostic technologies based on very fast DNA analysis, such as LAMP (Loop Mediated Isothermal Amplification) (e.g., Niu et al., 2011), are emerging that would be vitally effective in identifying new pests, thereby facilitating swift critical decision-making around containment and eradication. Work has now also advanced in the use of multiple stable isotopes to assess the natal origin of single insects as another decision-making tool. Unlike any other method, this can help determine whether the discovery of a threat species is part of an established population, including possible re-emergence of what was presumed to be an already ‘eradicated’ population, or that the discovery is in fact a new incursion (Holder et al., 2014).

Likewise, the ongoing use of metapopulation modeling using improved knowledge of pest population biology, dynamics and dispersal data will permit more targeted pasture surveillance systems to continue to be developed. Such work helps to resolve uncertainty about those ecosystem processes between introduction, full invasion and establishment (Born et al., 2004). In part, these advantages will be based on rapidly increasing computational power data-handling capability, including data-warehousing (Worner et al., 2014).

As mentioned earlier, eradication is particularly difficult with soil-dwelling species and is something that is often overlooked both socially and politically. Indeed, options for even acceptable local-site eradications are declining because of the abolition of use of various classes of pesticide, particularly those that persist in the ecosystem (Goldson et al., 2015).

Should eradication be deemed impossible, then expensive long-term control measures are required to be developed and implemented. However, before anything can be done, there is a need to understand the pest population dynamics which are often found to be markedly different from what is known of the species’ native range; this means dependence on overseas literature has limitations. Significantly, strategies for dealing with a new pest species, even in the pastoral environment, can mean resorting to the use of pesticides (e.g., seed treatments) that can completely disrupt finely balanced biological control systems (Goldson et al., 2015).

Looking further into the future and with suitable social consent, eradication based on techniques such as gene-editing e.g., CRISPR–Cas9 (Webber et al., 2016) and ‘Trojan gene’ techniques (Gemmell et al., 2013) have the potential to cause huge reductions in populations of pest species. For example, through meiotic-drive interventions, control along the lines of the sterile insect technique (SIT) have showed promise for managing mosquito vectors of disease (Burt, 2014). Ultimately, this type of technology could be coupled with uses of unmanned aerial vehicles (UAVs) for either surveillance or delivery of control technologies, such as is starting to be used for weed control (Torres-Sánchez et al., 2013). UAVs are already capable of scouting large swaths of land and could include the use of sequential pictures with a computer algorithm to automatically screen for the effects of unexpected invasive pest species.

## Conclusion

The confounding challenges of pasture biosecurity, as outlined in this contribution, has meant that the sector is less able to focus on this issue than the crop and horticultural sectors. In part this is because forage is neither consumed by humans nor exported.

A major constraint for pasture biosecurity is the lack of well-defined risk-species pathways, which are particularly afflicted by difficult-to-detect and difficult-to-identify hitchhiker species. This severely limits pre- and at-border opportunities for disinfestation measures. Moreover, eradication is often effectively impossible when commonly soil-dwelling life stages are involved.

While there is no silver bullet, opportunities for improving pasture biosecurity may include increased vigilance by farmers (e.g., via the pending GIA), plant resistance, more use of advanced population dispersal models and surveillance strategies, pheromone traps, and emerging genetic and isotope techniques to identify pests and their origins.

## Author Contributions

KA and SG conceived the topic and wrote substantial parts of the text. BB revised the text critically for accuracy and intellectual content. SG, KA, and BB all approved the version to be published and agreed to be accountable for all aspects of the work.

## Conflict of Interest Statement

The authors declare that the research was conducted in the absence of any commercial or financial relationships that could be construed as a potential conflict of interest.

## Footnotes

1 http://www.stats.govt.nz/browse_for_stats/industry_sectors/imports_and_exports/overseas-merchandise-trade-info-releases.aspx

2 http://www.stats.govt.nz/browse_for_stats/environment/environmental-reporting-series/environmental-indicators/home/land/land-cover.aspx

3 http://agpest.co.nz/

4 http://www.dairynz.co.nz/

5 http://www.beeflambnz.com/

6 https://www.far.org.nz/

7 http://www.gia.org.nz/

8 http://www.plantsentinel.org/

9 http://www.biologicalheritage.nz/home
